# The impact of high-intensity interval training on anxiety: a scoping review

**DOI:** 10.3389/fpsyt.2025.1515266

**Published:** 2025-02-19

**Authors:** Yidan Wang, Xiaotu Zhang, Ye Zhang, Hongshi Zhang

**Affiliations:** Department of Nursing, Changchun University of Chinese Medicine, Changchun, China

**Keywords:** HIIT, anxiety, mental health, mental disorders, high-intensity exercise

## Abstract

**Background:**

In recent years, high-intensity interval training (HIIT) has gained significant attention due to its efficient use of time. Studies have shown that engaging in regular physical activity can effectively reduce symptoms of anxiety. Given the potential side effects and limitations associated with pharmacological treatments for anxiety disorders, there is a growing interest in exploring non-pharmacological interventions. HIIT, as an alternative approach, offers a promising avenue for managing anxiety without relying on medication. However, the specific efficacy and applicability of HIIT for individuals diagnosed with anxiety disorders have not been systematically summarized in the literature. This scoping review aims to explore the effectiveness of HIIT as an intervention for improving anxiety symptoms, as well as its range of applicability, by synthesizing existing research.

**Methods:**

A scoping review methodology was employed to search databases including PubMed, Web of Science, EMbase, and the Cochrane Library. Studies were selected based on predefined criteria: randomized controlled trials (RCTs), cohort studies, and quasi-experimental designs focusing on the improvement of anxiety, using HIIT as the primary intervention. Exclusion criteria included studies with mixed interventions or populations with comorbid conditions. Two independent evaluators screened titles, abstracts, and full texts, extracted data using a pre-tested form, and resolved discrepancies through discussion to ensure consistency and accuracy.

**Results:**

A total of 541 articles were identified, of which 16 met the inclusion criteria for this review. The samples comprised various populations, including healthy subjects, overweight males, prostate cancer patients, etc. Twelve studies indicated that HIIT significantly improves anxiety symptoms, especially for individuals with lower baseline anxiety. The effect was less pronounced in those with higher baseline anxiety. Additionally, the studies suggest that variations in training protocols—such as frequency, intensity, and duration—may influence the extent to which HIIT improves anxiety symptoms.

**Conclusions:**

This study underscores the potential of High-Intensity Interval Training (HIIT) as an effective intervention for reducing anxiety symptoms, especially when tailored to individual baseline characteristics. Variations in training parameters such as frequency, intensity, and duration are critical factors in optimizing HIIT’s effectiveness for mental health benefits.HIIT shows promise as a valuable tool for managing anxiety, with the potential to significantly improve mental health outcomes when implemented with careful consideration of individual differences and protocol variations. This study provides a foundation for refining HIIT protocols and expanding their applicability across diverse populations.

## 1 Introduction

Anxiety disorders represent a significant and pervasive mental health issue globally, affecting millions of individuals and imposing a considerable burden on society. Empirical data reveals that anxiety disorders are among the most prevalent mental health conditions, with estimates suggesting that they affect around 18% of the global population (1). This high prevalence, combined with their chronic nature and frequent comorbidity with other mental health conditions, has led the World Health Organization (WHO) to rank anxiety disorders as the ninth leading cause of disability worldwide (2). Anxiety, characterized by an unpleasant emotional state with experiential, physiological, and behavioral components, serves as a vital warning mechanism for individuals (3). As defined in the Diagnostic and Statistical Manual of Mental Disorders (DSM-V), anxiety encompasses a range of diagnoses, including panic disorder, agoraphobia, generalized anxiety disorder, post-traumatic stress disorder (PTSD), social phobia, acute stress disorder, obsessive-compulsive disorder, and disorders related to medical conditions or substance use (4). Manifesting in symptoms such as sweating, chills, trembling, increased heart rate, hyperventilation, and a sense of impending doom, anxiety disorders can severely impact an individual’s quality of life (5).

Furthermore, research has demonstrated that persistent anxiety is associated with an elevated risk of cardiovascular diseases, with a risk ratio of 1.5 (6). In addition to cardiovascular diseases, anxiety has been linked to an increased risk of developing other physical illnesses, such as stroke, diabetes, arthritis, and lung diseases, compared to the general population (7). The mechanisms contributing to this higher comorbidity risk in populations with anxiety disorders may include unhealthy lifestyles, poor treatment adherence, and dysregulation of psychobiological stress systems. Therefore, early integration of physical health concerns into the treatment of anxiety disorders is crucial. Additionally, anxiety disorders can lead to unstable interpersonal relationships, functional impairment, and increased workplace absenteeism, resulting in substantial economic losses (8). Given these alarming statistics and the multifaceted impact of anxiety disorders, it is imperative to explore effective interventions, such as High-Intensity Interval Training (HIIT), to address this pressing public health issue.Reducing the substantial burden of disease caused by anxiety disorders, both for individuals and globally, can be most effectively achieved through timely and accurate disease detection, along with the administration of adequate treatment and the scaling up of interventions when necessary. Worldwide, the treatment rate for anxiety symptoms remains low, a challenge that is particularly pronounced in low-income countries but continues to be a pressing issue in high-income countries as well (9).

As mental health concerns like anxiety rise, while prescription medications and psychotherapy remain essential, current studies indicate that increased reliance on exercise, such as High-Intensity Interval Training (HIIT), can alleviate negative moods and reduce healthcare costs (10). HIIT’s effectiveness across various health parameters is supported by empirical evidence (11), and theoretical frameworks provide insights into its mechanisms.Theoretically, HIIT aligns with Social Cognitive Theory (SCT), which suggests that successful engagement in challenging activities like HIIT can enhance self-efficacy, leading to improved mental health outcomes, including reduced anxiety (12). The Theory of Planned Behavior (TPB) indicates that HIIT’s structured and time-efficient nature may enhance perceived behavioral control, promoting adherence and long-term engagement (13).Empirically, HIIT involves intensities eliciting ≥80-100% of peak heart rate, with “all-out” Sprint Interval Training (SIT) exceeding maximal oxygen uptake (14). Originally for endurance athletes, HIIT now benefits the general population, including previously sedentary individuals (15). Studies show HIIT significantly improves anxiety symptoms, especially in those with lower baseline anxiety levels (16), highlighting its potential as a therapeutic intervention for mental health.

Exercise training has been shown to effectively improve mood disorders in both humans and rodents, with recent studies indicating that exercise can ameliorate chronic stress-induced anxiety in mice by modulating RNA N6-methyladenosine (m6A) levels (17). Despite extensive literature on general exercise therapy for anxiety and depression (18), research specifically on High-Intensity Interval Training (HIIT) remains in its early stages, with only a limited number of studies exploring its effectiveness in alleviating anxiety symptoms. To address this gap, this scoping review aims to summarize existing systematic reviews related to HIIT and anxiety, understand its current role in anxiety research, identify gaps, and determine future research directions. By enhancing HIIT interventions for anxiety patients, this review seeks to guide clinical practice. The paper will introduce the research question, outline the scoping review methodology, compile and analyze relevant literature, and interpret findings within the broader context of mental health research, suggesting practical implications and avenues for future research. This structured approach ensures the research question is grounded in current knowledge while paving the way for innovative advancements in the field.

## 2 Materials and methods

This review has been carried out in accordance with the preferred reporting items for systematic reviews and meta-­analyses (PRISMA) statement (19) and PRISMA 2020 checklist (**Supplementary File 1**).

### 2.1 Research design

This scoping review will employ a systematic and rigorous methodology to synthesize the existing evidence on the impact of High-Intensity Interval Training (HIIT) on anxiety. The review will commence with an extensive search of electronic databases, including PubMed, Web of Science, Scopus, and Cochrane Library, to identify relevant studies. The search strategy will be developed using a combination of keywords related to HIIT, anxiety, and their interrelationship. Additionally, reference lists of retrieved articles and gray literature sources will be screened to ensure comprehensive coverage. Studies included in the review will be screened based on predefined inclusion and exclusion criteria, focusing on original research articles that investigate the relationship between HIIT and anxiety in humans. Data extraction will involve summarizing key characteristics of each study, including population demographics, study design, HIIT protocols, anxiety assessment tools, and main findings. The extracted data will be synthesized narratively, highlighting key trends and findings across studies. Furthermore, the review will examine the specific effects of HIIT on anxiety across different populations and settings, and explore potential mechanisms underlying the relationship between HIIT and anxiety. Finally, the review will identify research gaps and suggest future directions for research in this area, contributing to a more nuanced understanding of the impact of HIIT on anxiety and informing evidence-based practice and policy recommendations.

### 2.2 Search strategy

To identify relevant studies, we conducted an extensive search of the following electronic databases: PubMed, Web of Science, EMBASE, and the Cochrane Central Register of Controlled Trials (CENTRAL), as well as Chinese databases including CNKI (China National Knowledge Infrastructure), Wanfang Data, and VIP (Chongqing VIP Information). These databases were chosen to cover a broad spectrum of medical, psychological, and scientific literature. The search covered all available years from the inception of each database up to September 25, 2024, ensuring the inclusion of the most recent research.A combination of controlled MeSH terms and free text were used as descriptors, which were combined with the Boolean operators ‘AND’ and ‘OR’The following search terms were used: (((((anxiety [Title/Abstract]) OR (angst [Title/Abstract])) OR (nervousness [Title/Abstract])) OR (anxious [Title/Abstract])) AND (High Intensity Interval Training [Title/Abstract])) OR (HIIT [Title/Abstract]) OR (repeated sprint training [Title/Abstract]) OR (sprint interval training [Title/Abstract]) OR (High-Intensity Intermittent Exercise [Title/Abstract]) (**Supplementary File 2**).

To carry out this review, a protocol was created and registered in PROSPERO (international prospective register of systematic reviews) under the number: CRD4202230342.

### 2.3 Inclusion criteria

Inclusion criteria were determined according to the PCC (Population, Concept, Context) principle: (1) Participants: Studies were included if they involved human subjects who received High-Intensity Interval Training (HIIT) as a therapeutic intervention aimed at improving anxiety (20). (2) Concept: Studies that involve high-intensity exercises interspersed with recovery periods, including those defined as HIIT or Sprint Interval Training (SIT). High-intensity exercise is defined as achieving >85% of VO2peak, reaching 85% of HRmax, or utilizing equivalent perceived exertion-based methods. Studies must adopt an intervention design with a training period lasting >1 week and should exclusively focus on HIIT (21). Additionally, studies should include measures related to any of the following outcomes: (i) muscle function (strength or power), (ii) muscle mass, or (iii) physical performance (22). (3) Context: Studies conducted in any type of setting (e.g., laboratory, private clinic, rehabilitation center, hospital) where anxiety is the primary outcome measure were selected based on predefined criteria including randomized controlled trials (RCTs), cohort studies, and quasi-experimental designs (23). Physical improvements from HIIT enhance self-efficacy, body image, and confidence in daily activities, while also promoting better sleep and mood, all of which can positively impact anxiety levels. These outcomes serve as important secondary markers, providing insights into the broader mental health benefits and long-term effectiveness of HIIT (24–27).

### 2.4 Exclusion criteria

(1) Studies that have not undergone full peer review (e.g., conference proceedings, posters, published abstracts, non-peer-reviewed articles, proposed studies, theses, dissertations, reviews, commentaries, debates). (2) Duplicates of published literature. (3) Studies for which the full text is not available. (4) Animal experiments, reviews, letters to the editor, and similar publications. (5) Manuscripts written in languages other than English. Exclude studies where randomization is not applied to a control group, such as studies comparing two experimental groups without a control group. (6) Studies involving animal models (e.g., rodent models). (7) Studies where HIIT is combined with other interventions (e.g., cognitive-behavioral therapy, pharmacotherapy). No language restrictions were applied to ensure that potentially relevant studies were not excluded due to the language of publication. Only full-text articles that met the eligibility criteria were considered for inclusion.

### 2.5 Data items

Data extracted from each study included sample size, group descriptions, study design, analytical methods, and outcome data. Methodological quality was assessed using a modified 0–10 point PEDro scale (16). Primary outcome variables were defined as pre- and post-intervention self-report questionnaires [Beck Anxiety Inventory (BAI), State-Trait Anxiety Inventory (STAI), Hospital Anxiety and Depression Scale (HADS)], clinical assessment tools [Clinical Global Impression (CGI), Structured Clinical Interview for DSM Disorders (SCID)], physiological indicators (heart rate variability (HRV), galvanic skin response (GSR), cortisol levels), and behavioral indicators (task performance, self-reported behavioral changes). Due to the heterogeneity in inclusion criteria, interventions, assessment tools, and outcomes, a meta-analysis was not deemed appropriate. The reliability and validity of the scales used in this scoping review have been rigorously tested in previous studies. For instance, the Beck Anxiety Inventory (BAI), a 21-item self-report questionnaire designed to measure the severity of anxiety symptoms, has demonstrated high internal consistency (Cronbach’s α typically ranging from 0.90 to 0.94) and good test-retest reliability (28). The BAI is widely recognized for its strong psychometric properties and has been validated across various populations (29). Similarly, the State-Trait Anxiety Inventory (STAI), which includes two separate 20-item subscales for measuring state anxiety (STAI-S) and trait anxiety (STAI-T), has shown excellent reliability and validity in numerous studies (30). The STAI-S assesses transitory anxiety levels related to specific situations, while the STAI-T measures more stable, long-term anxiety tendencies. The Hospital Anxiety and Depression Scale (HADS), a 14-item scale with two subscales for anxiety and depression, has also been extensively validated (31). It is commonly used in clinical settings and has demonstrated good sensitivity and specificity for detecting anxiety and depressive disorders. For clinical assessment tools, the Clinical Global Impression (CGI) scale, which consists of three subscales—Severity, Improvement, and Efficacy Index—has been widely used and validated in psychiatric research (32). The CGI provides a global assessment of symptom severity and treatment response by clinicians. The Structured Clinical Interview for DSM Disorders (SCID) is a comprehensive diagnostic tool that has undergone rigorous testing for both reliability and validity (33). SCID is used to diagnose mental disorders according to DSM criteria and is considered a gold standard in clinical assessments.

### 2.6 Data extraction

**Figure 1:**
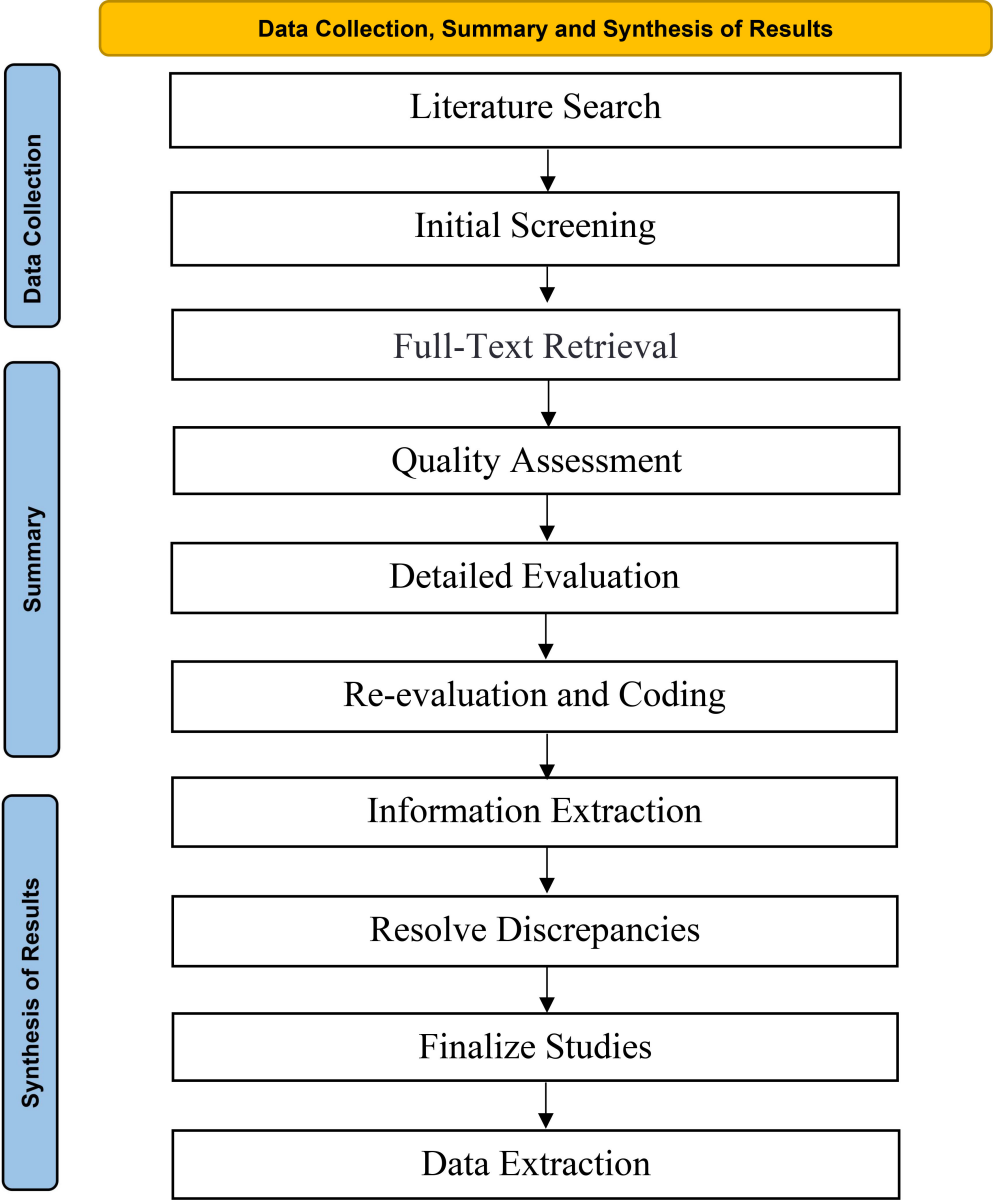
Data collection, summary and synthesis of results flow diagram.

Two authors independently conducted the literature search. After completing the search of each database and retrieving the papers, all studies were downloaded into a single reference list and imported into EndNote X9 software to remove duplicates. Titles and abstracts were then screened to determine if they met the inclusion criteria, with only studies involving HIIT selected for full-text retrieval. Subsequently, the two reviewers read and coded all included articles using the PEDro scale (34). Following this, the full texts were thoroughly evaluated against the inclusion and exclusion criteria, with final confirmation provided by the first author and the corresponding author. After this quality assessment, the same reviewers read and coded each study, evaluating the following moderators: study design [randomized controlled trial (RCT), controlled trial (CT), or uncontrolled trial (UCT)], whether HIIT was used alone or in combination, and outcome variables. Additionally, detailed participant descriptions and training program variables were extracted. Any discrepancies between reviewers were resolved during consensus meetings, with the consultation of a third researcher when necessary, to finalize the list of studies meeting the criteria. Data extraction was performed using a standardized form and cross-checked for accuracy (see the Data extraction flow diagram in **Figure 1**).

## 3 Results

### 3.1 Results of the literature search

**Figure 2:**
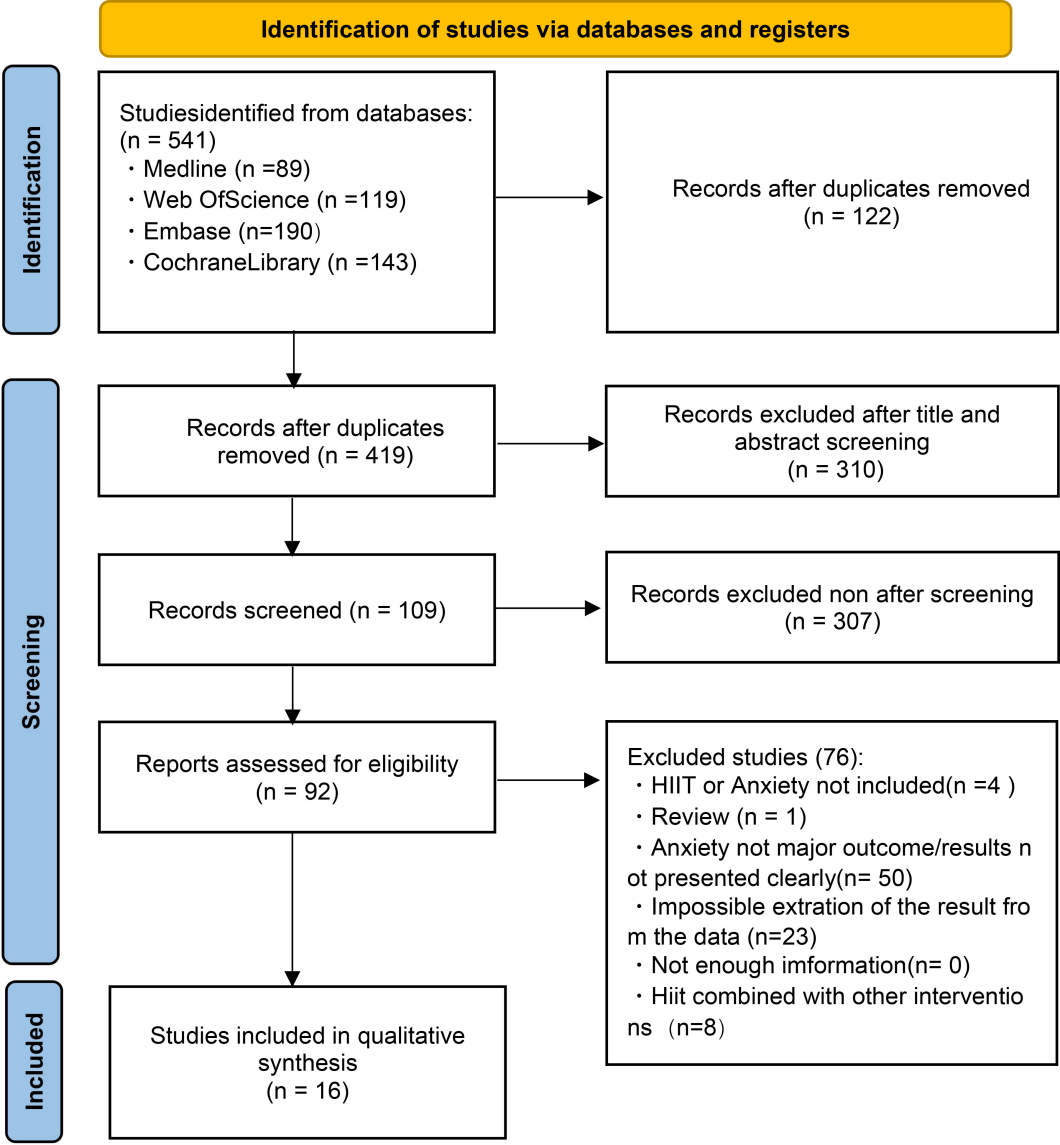
Flow diagram of trials selection process.

The search strategy identified 541 records across four electronic databases. After removing duplicates, 416 records remained. Of these, 302 records were excluded based on title and abstract screening. The full texts of the remaining 115 articles were assessed, and ultimately, 16 studies meeting the inclusion criteria were included (see the PRISMA flow diagram in **Figure 2**).

### 3.2 Characteristics of included studies

The characteristics of these studies are outlined in **Table 1**. All included studies were published between 2005 and 2024. Specifically, publications were made in 2005 (n = 1), 2018 (n = 2), 2019 (n = 2), 2020 (n = 4), 2021 (n = 2), 2022 (n = 2), 2023 (n = 2), and 2024 (n = 1). Studies were conducted in eleven different countries or regions, with one study from Spain, two studies from Brazil, one study from Ireland, one study from the U.S.A., three studies from Canada, one study from Australia, one study from China, two studies from Germany, two studies from Tunisia, one study from Italy, and one study from Taiwan. And Canada contributed the most studies (n = 3). Four studies originated from Europe, two studies came from North America, two studies were from Asia, one study was from Oceania, one study was from South America, and one study was from Africa. The total sample size across all studies was 570 participants, with individual study participant numbers ranging from 12 to 67. Three studies included only female participants, five studies included only male participants, and eight studies included both male and female participants. The samples comprised various populations, including healthy subjects (n = 6) (35, 36, 38, 41, 44, 49), overweight males (n = 1) (37), prostate cancer patients (n = 1) (39), sedentary individuals (n = 1) (40), asthma patients (n = 1) (42), women with polycystic ovary syndrome (PCOS) (n = 1) (43), panic disorder patients (n = 1) (45), generalized anxiety disorder patients (n = 1) (46), soccer players (n = 2) (47, 48), and patients attending a psychiatric day care unit (n = 1) (50). Three studies involved participants diagnosed with anxiety and related affective disorders (45, 46, 50). Most studies adopted a randomized controlled trial (RCT) design (n = 10), while others included observational cohort studies (n = 3), pilot studies (n = 1), longitudinal studies (n = 1), and prospective studies (n = 1).

**Table 1:** Characteristics of included studies.

Article		Country	Population	Study design	Exercise protocol classification(s)	Exercise protocol classification(s)	Intervention characteristics						Outcome(s)	PEDro score
							Duration (weeks)	Total sessions	Exercise protocol	Exercise intensity	Adherence/ Compliance/ Attendance	Adverse events		
1	Borrega-Mouquinho et al. (35)	Spain	67 healthy adults (M=22, F=45;Mage=45y);HIIT group Mean (n=36), MIT group Mean (n=31)	RCT	The study was registered in the Australian New Zealand Clinical Trials Registry	HIIT;MIT	6 weeks(6 days per week)	36	HIIT:10-minute warm-up, followed by high-intensity exercises (10-12 sets of 30-90 seconds with 15-60 seconds rest between sets) performed at 7-9 on the RPE scale (corresponding to 85-95% HRmax), and concluded with a cooldown lasting 30-40 seconds.	6–9 of RPE(rate of perceived exertion)	67 started, 53 finished.	Not reported	symptoms ofdepression , stress, state of anxiety, and resilience	9
2	Chavan et al. (36)	Brazil	23 healthy adult women(mean ± SD age, 22 ± 4 yr; mean ± SD body mass index (BMI), 24 ± 4 kg·m−2)	Observational Cohort Study	No	HIIT	completed a cycle ergometer HIIT session in the menstrual, follicular, and luteal phases of the menstrual cycle(total 7-11days)		2-minute warm-up and cool-down, 20-minute sessions with 60-second intervals followed by 60-second active recovery, and a 5-minute post-exercise seated assessment.	achieved at least two of the following criteria: i) a peak HR of at least 90% of age predicted maximal HR (based on 220 − age), ii) RPE ofat least 8 (on a0–10 scale), and iii) respiratory exchange ratio ofat least 1.15.	23 started, 22 finished.	reported higher pain, water retention, behavior change (alterations in actions, habits, etc.),	menstrual cycle–related symptoms, anxiety, mood, HR, arousal, affective valence, and motivation.	5
3	De Sousa et al. (37)	Brazil	25 middle-aged overweight men (aged 30–50 years; BMI ≥25 kg/m2)	RCT	No	MICT;HIIT	8 weeks (three times per week)	24	repeated 200-m sprints (10×20 m) interspersed with 1-min bouts of passive recovery	run at 85% (weeks 1–2), 90% (weeks 3–6), 95% (week 7), or 100% (week 8) maximum velocity	100% adherence	Not reported	HRQoL, depression, and anxiety levels	7
4	Herring et al. (38)	Ireland	38 young adult males[(BMI<30 kg m−2) males aged 18-35y]	Observational Cohort Study	No	SIT	3 weeks(three times per week)	9	consisting of 4–6 maximal 30-second cycle sprints with 4 minutes of active recovery between sprints, preceded by a 5-minute warm-up and followed by a 3-minute cool-down	RPE of 20	100% adherence	Not reported	State anxiety	6
5	Kang et al. (39)	U.S.A	52 patients with prostate cancer ; interval training(n=26), usual care (UC)(n=26)	RCT	The trial was registered with clinicaltrial.gov	HIIT	12 weeks(three times per week)	36	5-minute warm-up, followed by 2 to 8 cycles of 2-minute high-intensity walking or jogging and 2-minute active recovery, concluding with a 5-minute cooldown and 5 minutes of lower body muscle stretching.	at 85% to 95% of VOzpeak	52 started, 50 finished.	HIIT in our study did not affect patient-reported physical functioning or physical symptoms (eg urinary incontinence, bowel functioning, sexual functioning, nausea, dyspnea).	anxiety, fear of cancer progression, quality of life and psychosocial outcomes	8
6	Lucibello et al. (40)	Canada	61 low active young adults (Mage ± SD = 19.8 ± 2.2 years, 63% female);HIIT (n = 28), PLACEBO (n = 32).	RCT	The trial is registered at Clinicaltrial.gov	HIIT	9 weels (three times per week)	27	3-minute warm-up and a 2-minute cool-down, followed by 20 minutes of intervals alternating between 1-minute sprints, and 1 minute of active rest.	90–95% of maximum HR and 80% of maximum W, and 1 minute of active rest at 30% of maximum W.	60 started, 4 6 finished.	Not reported	anthropomorphic measures, psychological (surveys) and physiological (blood sample draw) indicators of anxiety and depressive symptoms, and a VO2peak test	7
7	Mason et al. (41)	Canada	56 undergraduate students or community members (male=11, female=45;agesd 18-65 years; MICT (n=20) ,SIT (n=16)	RCT	No	SIT;MICT	1 week	7	2-minute warm-up, followed by three 20-second sprints, separated by approximately 2 minutes of low intensity cycling for active recovery.	above 18 RPE and 85% age-adjusted HRmax,	100% adherence	Not reported	anxiety sensitivity	6
8	O’Neill et al. (42)	Canada	20 adults with asthma (ages of 18–44 years,n = 20, 11 of 20 were female)	Observational Cohort Study	No	HIIT	6 weeks(3 days per week)	18	5-minute warm-up at 25 W for 1 minute, 90% peak power output for 1 minute, repeated 10 times.	10% peak power output for 1 min, 90% peak power output for 1 min	100% adherence	Not reported	anxiety	3
9	Patten et al. (43)	Australia	29 polycystic ovary syndrome women (aged 18–45 years); MICT (n = 15), HIIT = (n=14)	two-arm RCT	The trial was registered in the Australian New Zealand Clinical Trials Registry	MICT;HIIT	12 weeks(three times per week)	36	two sessions of 12 × 1 min intervals , interspersed with 1 min of active recovery at a light load; and one session of 8 × 4 min intervals, interspersed with a 2 min light load, active recovery.	two sessions at 90–100%HRpeak (~ 10 METs) at ;one session at 90–95%HRpeak (~ 8 METs)	83 started, 29 finished.	Not reported	Mental health and health-related quality of life	7
10	Philippot et al. (44)	China	30 students (aged 18–25 years, female=25, male= 3); HIIT (n = 13), Control (n = 15)	RCT	No	HIIT	4 weeks(three times per week)	12	Alternating between High intensity exercises (30 s) and active recovery exercises (30 s) to reach 10min	≥6 RPE	30 started, 2 8 finished.	HIIT did not produce any adverse effects in the present study	psychological symptoms	9
11	plag et al. (45)	Germany	12 patients (9 female, 3 male) with a primary diagnosis of PD (Mean age was 32.7 years)	A pilot study	No	HIIT	12-day		10 high-intensive 1-minute intervals separated by 1-minute intervals with moderate to low intensity	77% to 95% maximumheart rate	100% adherence	Not reported	PD severity,Barriers to exercise and self-efficacy,endurance performance,subjective level of exhaustion	4
12	plag et al. (46)	Germany	33 patients with generalized anxiety disorder (GAD)(Female=24, Male=9)	parallel-group, assessor-blinded RCT	No	HIIT;LIT	12-day		trained every other day for 12 days on a bicycle ergometer, with each 20-minute session consisting of alternating one-minute intervals preceded and followed by a five-minute warm-up and cool-down period, respectively.	at 77–95% HRmax and less than 70% HRmax	Adherence rate was 82 % for HIIT and 94 % % for the control group	Not reported	Anxiety, comorbid depression, stress-related bodily symptoms and perceived control over anxiety related stimuli (PC)	7
13	selmi et al. (47)	Tunisia	30 elite soccer players (age: 17.8 ± 0.9 years); RST-G(n = 15), control group (n = 15)	RCT	No	repeated sprint (RS) training	6 weeks(3 days per week)	18	short bouts of RS 2–3 sets of 5–6 × 30 to 40 m interspersed with 20 s of active recovery	100%	100% adherence	Not reported	somatic anxiety (SA), cognitive anxiety (CA), self-confidence (SC), rating of perceived exertion (RPE) and repeated sprint ability (RSA)	5
14	Selmi et al. (48)	Tunisia	38 highly trained male athletes (aged 18.9 ± 0.5 years); aerobic and speed training group (n = 20), active control group(n=18)	RCT	No	aerobic and speed training group	6 weeks(5 days per week)	30	15-minute standardized warm-up, followed by 5 minutes of dynamic stretching, and then completed a repeated sprint ability test consisting of 6 × (20 + 20 m) runs with 20-second passive recovery intervals.	at 110–120% of the final velocity achieved at the end of the test.	100% adherence	None reported any training- or test-related injury	aerobic power, repeated sprint ability and somatic anxiety, cognitive anxiety, and self-confidence	6
15	Viana et al. (49)	Italy	36 healthy women; HIIT (n = 18) , SIT (n = 18)	longitudinal study	No	SIT;HIIT	8 weeks (three times per week)	24	SIT:a warm-up of 5 min, following by four repeated 30 s all-out cycling efforts, alternated with 4 min of passive recovery or light cycling with no load lasted 23 min. HIIT:f a warm-up of 5 min , followed by four repeated 4 min efforts, alternated with 3 min of recovery lasted 33 min	SIT: frequency ≥60 rpm HIIT: at 90% to 95% of HRpeak	36 started, 34 finished.	Not reported	depressive and anxious symptoms	5
16	Wu et al. (50)	Taiwan	20 patients attending a psychiatric day care unit	prospective study	No	HIIT	8 weeks (three times per week)	24	5-minute warm-up period, followed by a 15-minute course of HIIT, and then a 5-minute period of stretching last 25 minutes	above 95% of maximum HR	20 started, 18 finished.	Not reported	physical and mental health	5

### 3.3 Intervention characteristics

In the 16 HIIT protocols applied in the reviewed studies, 12 (75%) were classified as HIIT, 3 (18.8%) were classified as SIT, and 1 (6.3%) was classified as aerobic and speed training. The classification of these protocols is based on the definitions and criteria explicitly described by the authors within their respective studies. There was considerable variation among the interval training protocols; for example, the duration and intensity of warm-up, cool-down, work intervals, and rest periods differed significantly. Among the sixteen studies, the warm-up protocols prior to initiating HIIT varied as follows: five studies included a 5-minute warm-up; two studies implemented a 2-minute warm-up; one study conducted a 3-minute warm-up; one study performed a 10-minute warm-up; and one study carried out a 15-minute warm-up. Notably, six studies did not provide any description of their warm-up procedures. Regarding training intervals, two studies set specific exercise distances as their training targets: one aimed for 200 meters (37) and the other for 240 meters (48). The remaining fourteen studies targeted specific training durations. Among these, three studies had single-session training durations of less than 30 seconds (16, 38, 41), while six studies set a single-session training duration of 1 minute.

Following each bout of high-intensity exercise, ten studies incorporated rest intervals of 1 minute or less, one study designed a 2-minute rest interval, one study specified a 3-minute rest interval, and another study implemented a 4-minute rest interval. One additional study also used a 1-minute rest interval, whereas one study did not specify the duration of rest intervals.The most commonly reported interval training protocol was the HIIT scheme, which included a 5-minute warm-up, 1 minute of high-intensity exercise, followed by a 60-second recovery period (n = 4).

Regarding the mode of exercise in the intervention groups, 7 studies used cycle ergometers, 1 study used treadmills, 2 studies involved bodyweight resistance training, and 5 studies included repeated running or sprint training. The articles reported psychological and psychiatric outcome assessments, primarily focusing on anxiety. The training durations mostly ranged from 1 to 8 weeks, with 6 or 8 weeks being the most common intervention lengths. The frequency of exercise was predominantly three times per week. The control groups typically engaged in moderate aerobic exercise or received no specific intervention. Six studies reported 100% adherence among their participants, indicating that all participants in these studies strictly followed the designated protocols throughout the intervention period.

### 3.4 Measurement of anxiety

The primary outcome measure was anxiety level. All studies used a variety of assessment tools to evaluate anxiety levels, including the State-Trait Anxiety Inventory (STAI) (n=3), Beck Anxiety Inventory (BAI) (n=4), Memorial Anxiety Scale for Prostate Cancer (n=1), Anxiety Sensitivity Index-3 (ASI-3) (n=1), Depression, Anxiety, and Stress Scale (DASS-21) (n=2), Panic and Agoraphobia Scale (PAS) (n=1), Clinical Global Impression (CGI) (n=1), Hamilton Anxiety Rating Scale (n=1), Anxiety Control Questionnaire-Revised (ACQ-R) (n=1), and Competitive State Anxiety Inventory (CSAI-2) (n=2). Overall, the Beck Anxiety Inventory (BAI) (28) was the most commonly used measure.

Kang’s study assessed PCa-specific anxiety by the Memorial Anxiety Scale for Prostate Cancer (MAX-PC), which consists of a total score and 3 subscales assessing PCa anxiety, prostate specific antigen (PSA)-related anxiety and fear of recurrence (39). In their respective studies, Mason and O’Neill chose the Anxiety Sensitivity Index-3 (ASI-3) as the measurement tool for undergraduate students and community members, as well as for individuals with asthma. The reason for this choice may be that the ASI-3 can directly measure the core variable of anxiety sensitivity rather than general anxiety levels. This allows for a better understanding of how Sprint Interval Training (SIT) and Moderate Intensity Continuous Training (MICT) impact anxiety sensitivity. Additionally, the ASI-3 provides sufficient sensitivity to detect the effects of the interventions, thereby offering robust support for or against the study hypotheses.

Positive effect studies demonstrated significant reductions in anxiety sensitivity and asthma-specific anxiety following the six-week HIIT intervention compared to the control groups. These improvements were evident across various age groups, genders, and health statuses. In contrast, studies with negative results did not show a significant difference in anxiety sensitivity or asthma-specific anxiety between the HIIT group and the control group.

Among the 16 included studies, 12 reported that HIIT could improve anxiety levels in participants. One study indicated that three weeks of SIT could enhance anxiety and worry in young adult males. The training protocol included 4 to 6 maximal 30-second cycling sprints, with each sprint followed by 4 minutes of active recovery. Participants began the session with a 5-minute warm-up and ended with a 3-minute cool-down period (38).

Twelve weeks of HIIT reduced prostate cancer-specific anxiety (total score and fear of progression), hormonal dysfunction, and general psychosocial outcomes (stress, fatigue, and self-esteem), primarily attributed to a moderate to large reduction in fear of cancer progression (d = -0.67). The training protocol included 5-minute warm-up, followed by 2 to 8 cycles of 2-minute high-intensity walking or jogging and 2-minute active recovery, concluding with a 5-minute cooldown and 5 minutes of lower body muscle stretching (39).

Another study demonstrated that six weeks of HIIT (35) reduced anxiety, stress, and depression while increasing resilience in the general population during COVID-19 confinement, potentially linked to the activation of endogenous opioid substances that alleviate stress (51). O’Neill’s study (42) showed that a six-week HIIT intervention reduced anxiety sensitivity and asthma-specific anxiety in adults with subclinical anxiety levels, with these reductions being independent of gender, supporting the prescription of high-intensity interval training. For patients with existing anxiety symptoms, the intervention effects of HIIT were twice as large compared to low-intensity training, suggesting it could serve as a first-line complementary treatment for this condition (46).

Four studies indicated that HIIT did not improve anxiety. One study found that moderate-intensity continuous training (MICT) could reduce anxiety levels in middle-aged overweight men, while the anxiety levels in the HIIT group showed no significant changes. The observed differences in anxiety reduction between MICT and HIIT groups may be attributed to variations in perceived exertion, exercise adherence, physiological adaptations, individual variability, and measurement sensitivity. These factors highlight the importance of considering both the type and intensity of exercise when designing interventions aimed at improving mental health outcomes in specific populations.

Additionally, De Sousa (37) and Lucibello (40) noted that nine weeks of HIIT training did not reduce anxiety symptoms, depressive symptoms, or inflammatory states. From the perspective of participant characteristics, individual differences in baseline health status, mental health condition, and personal preferences may influence each participant’s response to different types of training.

Middle-aged overweight men might benefit more from the steady and manageable challenge of Moderate Intensity Continuous Training (MICT) compared to the intense demands of High-Intensity Interval Training (HIIT) (52, 53).

For people with asthma, anxiety symptom severity moderated cardiorespiratory improvements from HIIT such that individuals with low anxiety at baseline experienced greater improvements than those with higher anxiety. This points to a novel non-responder phenotype based on anxiety status.

Despite Lucibello’s 9-week High-Intensity Interval Training (HIIT) program for young adults not reducing symptoms of anxiety, depression, or inflammation status, the severity of anxiety symptoms can moderate improvements in cardiorespiratory fitness from HIIT. Specifically, individuals with lower baseline anxiety levels exhibited greater improvements compared to those with higher anxiety levels. This points to a novel non-responder phenotype based on anxiety status (40).

A randomized controlled trial examining the impact of HIIT on students’ psychological states during the COVID-19 pandemic indicated that both HIIT and moderate-intensity training (MIT) could reduce stress, anxiety, and depression levels, while also enhancing psychological resilience. However, the improvement in depression appeared to be greater in the HIIT group compared to the MIT group.

These variations highlight the need for further investigation into the factors influencing the efficacy of HIIT interventions, such as individual differences in exercise tolerance, genetic backgrounds, lifestyle, and psychological states.

The observed variations in response to High-Intensity Interval Training (HIIT) among participants highlight the need for a deeper understanding of several influencing factors. These variations can be attributed to individual differences in exercise tolerance, genetic backgrounds, lifestyle factors, and psychological states (54). Participants with higher baseline fitness levels may tolerate and benefit more from HIIT compared to those with lower fitness levels. Genetic factors play a crucial role in determining how individuals respond to exercise. Some individuals may have genetic profiles that predispose them to better adaptation to high-intensity training, while others might not respond as positively (55).

Lifestyle encompasses various aspects such as diet, sleep patterns, alcohol consumption, smoking status, and overall physical activity levels outside of the intervention (56). Participants with healthier lifestyles—such as balanced diets, adequate sleep, and regular physical activity—may see more pronounced benefits from HIIT. Psychological states include mental health conditions, stress levels, motivation, and personal attitudes towards exercise. Individuals with pre-existing anxiety or depressive symptoms might find it more challenging to engage fully in HIIT due to increased psychological stress (20).

## 4 Discussion

### 4.1 Summary of findings

This scoping review investigated the application of HIIT in managing anxiety, summarizing findings from 16 eligible studies that reported outcomes related to anxiety associated with HIIT. The review provides a comprehensive perspective, aiding researchers and clinicians in understanding the potential of HIIT for anxiety management.While the results of the reviewed studies were mixed, a synthesis of multiple relevant studies revealed the value of HIIT in reducing anxiety levels, some studies show no significant effects. Most studies reported HIIT significantly decreased anxiety scores, with reductions averaging 7 to 10 points on standardized measurement tools such as the Beck Anxiety Inventory (BAI) and State-Trait Anxiety Inventory (STAI), indicating clinically meaningful changes. Improvements in at least one measure of muscle function or physical performance following HIIT interventions, indicating that HIIT may benefit not only mental health but also physical function. Despite the high intensity of HIIT, overall adherence was good, with an average attendance rate of 85%, reflecting the feasibility of HIIT as an intervention method. Additionally, this study focused on the impact of HIIT alone on anxiety and did not include other interventions combined with HIIT. This choice emphasizes assessing the potential therapeutic effects of HIIT itself on anxiety symptoms rather than the effects of combined interventions (57), which might have led to an underestimation of the potential benefits of integrated intervention strategies.

Some studies indicate that short-term HIIT programs, such as those lasting 4-6 weeks, can improve cardiovascular health and metabolic markers (48). However, the positive effects on mental health might require a longer duration to become evident (58). In contrast, HIIT programs that span several months may have a more pronounced positive impact on mental well-being (59). A defining characteristic of HIIT is its high-intensity interval pattern; however, the standard for what constitutes “high-intensity” varies across studies. Generally, HIIT intensity ranges from 80% to 95% of maximum heart rate. In this study, HIIT intensity was set above 85% of maximum heart rate, which falls within the typical range for high-intensity exercise (60). High-intensity training may lead to higher physiological stress responses, potentially affecting psychological states. However, it could also cause excessive stress for some individuals, which might be counterproductive to mental health (61). In comparison, moderate-intensity HIIT may be more suitable for beginners and easier to sustain (59). Appropriately adjusting the intensity of training may enhance the effectiveness of the regimen.

HIIT may influence anxiety through mechanisms such as endorphin release, improved cardiovascular fitness, and reduced inflammation, which are all associated with improved mental health. Physiologically, HIIT enhances cardiorespiratory fitness by improving cardiac output and circulation, positively impacting the nervous system and alleviating anxiety symptoms (57). It increases heart rate variability, which is associated with better autonomic nervous system regulation, thereby contributing to reduced anxiety levels (62). Additionally, high-intensity exercise promotes the release of endorphins, natural pain-relieving substances that enhance mood and decrease feelings of anxiety (63). HIIT may also help regulate cortisol levels, a hormone linked to stress responses, where elevated levels are associated with anxiety symptoms (64). Studies suggest that HIIT can lower inflammatory markers in the body, such as C-reactive protein (CRP), helping to reduce chronic low-grade inflammation, which positively influences anxiety (65). From a psychological perspective, HIIT boosts participants’ sense of self-efficacy by completing challenging tasks, making them more confident and better equipped to handle daily stressors (66). During exercise, attention shifts from anxiety sources to the activity itself, providing temporary relief from anxious feelings (64) and promoting cognitive improvements. Furthermore, the sense of accomplishment and enjoyment derived from HIIT contributes to increased positive emotions and decreased negative emotions, thus alleviating anxiety (67). Long-term engagement in HIIT can enhance coping strategies, enabling individuals to adopt more proactive and effective approaches to managing their anxiety (68).

### 4.2 Strengths and limitations

This study employed a scoping review methodology to systematically collect and analyze relevant literature on the impact of HIIT on anxiety, ensuring comprehensiveness and representativeness of the research (69). Rigorous literature screening criteria were applied to ensure the quality of the included studies, enhancing the reliability of the findings (70). This review not only evaluated the direct effects of HIIT on anxiety symptoms but also explored how baseline anxiety levels influenced the effectiveness of the training, providing a multi-dimensional analysis. The use of standardized psychological measurement tools, such as the Beck Anxiety Inventory (BAI) and the State-Trait Anxiety Inventory (STAI), ensured the comparability and scientific rigor of the study results.

Despite including multiple studies, several individual studies had small sample sizes. This issue significantly impacts the generalizability and robustness of the findings, as smaller samples are more susceptible to bias and variability, which can lead to less reliable results (57). Studies with small sample sizes often lack the diversity needed to represent broader populations accurately. Random fluctuations in participant characteristics or responses can disproportionately influence the results, leading to overestimation or underestimation of the actual impact of HIIT on anxiety. The duration of HIIT training in the reviewed studies was relatively short (typically around six weeks), which may not fully capture the long-term impact of HIIT on anxiety (58). Additionally, the HIIT protocols varied across studies in terms of training frequency, intensity, and duration, potentially leading to heterogeneity in the results (71). Additionally, small samples are more prone to selection bias, where the characteristics of participants might not be representative of the target population, further compromising the reliability of the outcomes. When small-sample studies are included in meta-analyses or systematic reviews, the overall conclusions drawn from these analyses can be skewed. Furthermore, the studies did not adequately address the impact of factors such as social support on anxiety symptoms, which may significantly influence the effectiveness of HIIT interventions on mental health (72). While this review examined the influence of baseline anxiety levels, other individual differences—such as genetic background and lifestyle habits—may also affect the efficacy of HIIT (69). Therefore, future research should aim for larger, more diverse samples to enhance the robustness and generalizability of findings.

Furthermore, the potential role of social support in enhancing the effectiveness of HIIT should not be underestimated. Social support, including encouragement from trainers, companionship from fellow participants, and reinforcement from family and friends, can significantly influence adherence to exercise regimens and boost motivation (73). Participants who receive robust social support are more likely to maintain consistent attendance and push through the challenging aspects of HIIT, leading to better psychological outcomes and sustained reductions in anxiety. Moreover, social interactions within group settings can foster a sense of community and belonging, which can directly alleviate feelings of isolation and improve mental health. Conversely, a lack of social support may result in decreased motivation, higher dropout rates, and less favorable outcomes. Therefore, future studies should consider integrating measures of social support into their analyses to fully understand its impact on the effectiveness of HIIT interventions (74).

## 5 Conclusions

This scoping review highlights the effectiveness of high-intensity interval training (HIIT) in alleviating anxiety symptoms, particularly among individuals with lower initial levels of anxiety. The clinical utility of HIIT as a therapeutic approach is noteworthy, offering a valuable option for those who favor treatments that do not rely on medication. By personalizing HIIT programs to match individual anxiety levels and considering longer training periods, sustained improvements over time can be achieved. Integrating HIIT with psychological therapies like cognitive behavioral therapy (CBT) and fostering social support significantly enhances outcomes for anxiety management, making it a robust, non-pharmacological tool that appeals to patients seeking alternatives beyond traditional drug-based interventions.

## 6 Practical recommendations

Future research should include extended follow-up periods to assess the enduring effects of HIIT on anxiety and focus on large-scale clinical trials using validated methods to better understand its efficacy and dose-response relationship. To maximize benefits, healthcare providers are encouraged to tailor HIIT programs to individual needs, consider longer training durations, and integrate HIIT with psychological therapies such as CBT. Fostering social support and creating environments that encourage adherence to HIIT programs can further enhance treatment outcomes, providing diverse and empowering pathways for individuals to improve their mental well-being.
