# Supplementary material for: The impact of high-intensity interval training on anxiety: a scoping review
Source: Front Psychiatry. 2025 Feb 19;16:1515266. doi: 10.3389/fpsyt.2025.1515266 (PMC11880788; doi:10.3389/fpsyt.2025.1515266)
Supplement: Supplementary file 2 [file SupplementaryFile2.docx]

**Search strategy**

(((((anxiety [Title/Abstract]) OR (angst [Title/Abstract])) OR (nervousness [Title/Abstract])) OR (anxious [Title/Abstract])) AND (High Intensity Interval Training [Title/Abstract])) OR (HIIT [Title/Abstract]) OR (repeated sprint training [Title/Abstract]) OR (sprint interval training [Title/Abstract]) OR (High-Intensity Intermittent Exercise [Title/Abstract]).

**1.Medline (n=89)**

(((((HIIT[Title/Abstract]) OR (repeated sprint training[Title/Abstract])) OR (sprint interval training[Title/Abstract])) OR (High-Intensity Interval Training[Title/Abstract])) OR (High-Intensity Intermittent Exercise[Title/Abstract])) AND ((((anxiety[Title/Abstract]) OR (angst[Title/Abstract])) OR (Nervousness[Title/Abstract])) OR (anxious[Title/Abstract]))

ENDNOTE


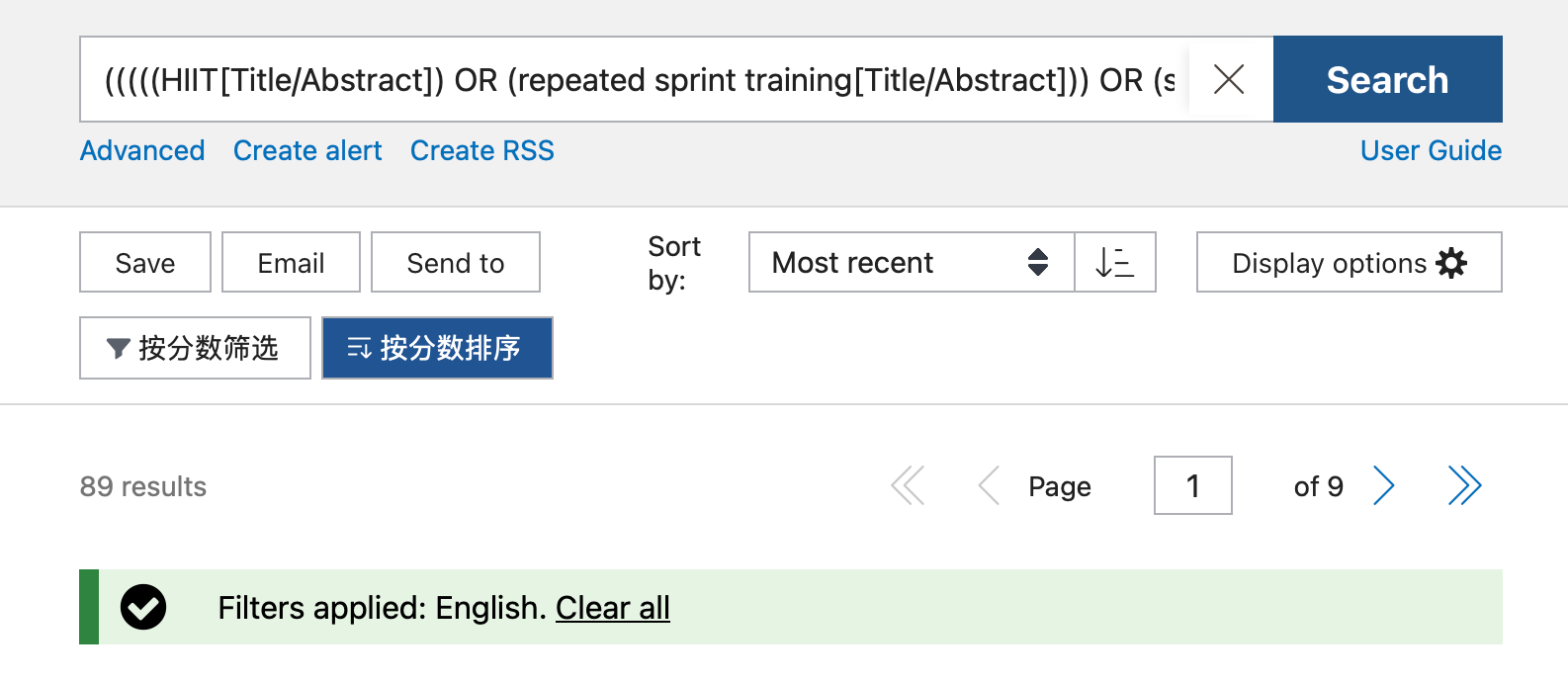


**2.Web Of Science (n =119)**

<https://webofscience.clarivate.cn/wos/woscc/summary/de52e054-37a8-4b98-a462-a44047502920-010b42bf37/relevance/1>

((((TS=(HIIT)) OR TS=(repeated sprint training)) OR TS=(sprint interval training)) OR TS=(High-Intensity Interval Training)) OR TS=(High-Intensity Intermittent Exercise) 8689

(((TS=(anxiety)) OR TS=(angst)) OR TS=(Nervousness)) OR TS=(anxious) 267,848

**(#1) AND #2 120**

**ENGLISH REFINED**

1. **Embase（n=190）**

Session Results

.......................................................

No. Query Results Results Date

#3. #1 AND #2 190 3 Oct 2024

#2. (anxiety OR angst OR nervousness OR 544,134 3 Oct 2024

anxious:ta,ab) AND [english]/lim

#1. (hiit OR 'high-intensity interval training' OR 5,179 3 Oct 2024

'repeated sprint training' OR 'sprint interval

training' OR 'high-intensity interval

training':ta,ab) AND [embase]/lim AND

[english]/lim


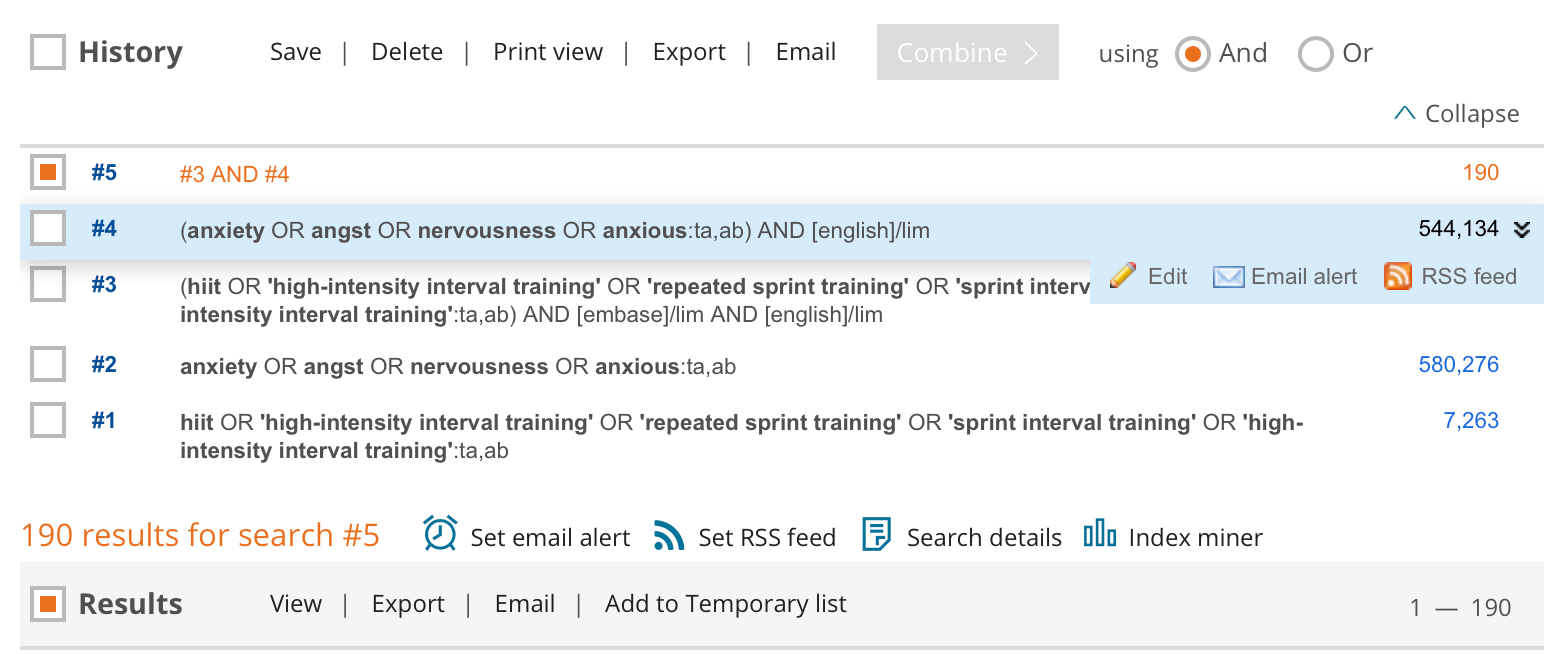


**4.CochraneLibrary (n =143)**

Search Name:

Date Run: 24/09/2024 16:54:41

Comment:

#1 MeSH descriptor: [High-Intensity Interval Training] explode all trees

#2 (HIIT OR epeated sprint training OR sprint interval training OR High-Intensity Interval Training OR High-Intensity Intermittent Exercise):ti,ab,kw (Word variations have been searched)

#3 #1 OR #2

#4 MeSH descriptor: [Anxiety] explode all trees

#5 (angst OR Nervousness OR anxious):ti,ab,kw

#6 #4 OR #5

# 3 AND #6


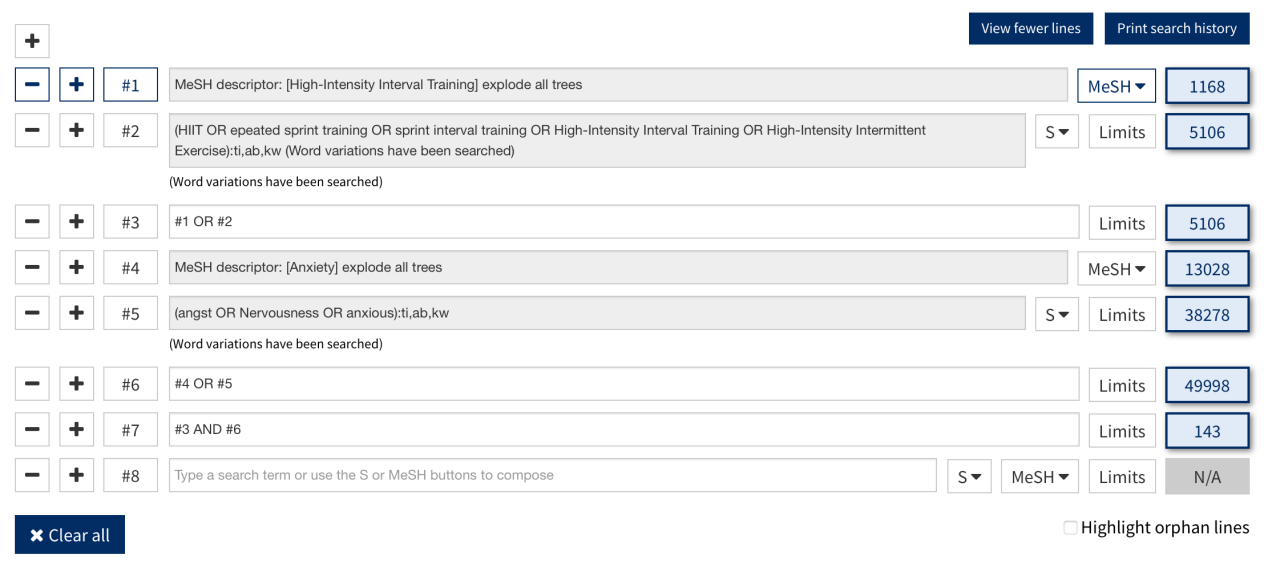


**TOTAL: 541 records**
